# Supplementary material for: Effects of bamlanivimab alone or in combination with etesevimab on subsequent hospitalization and mortality in outpatients with COVID-19: a systematic review and meta-analysis
Source: PeerJ. 2023 May 8;11:e15344. doi: 10.7717/peerj.15344 (PMC10174063; doi:10.7717/peerj.15344)
Supplement: Supplemental Information 2 [file peerj-11-15344-s002.zip › Supplementary file 1.docx]

**Supplementary file 1. Complete search strategy of our systematic review.**

# Pubmed

Search Strategy:

1 (covid 19 or covid-19).

2 "coronavirus disease 2019"

3 SARS-CoV-2

4 or/1-3

5 exp bamlanivimab

6 monoclonal antibody

7 etesevimab

8 or/5-7

9 4 and 8

((covid 19 or covid-19) OR “coronavirus disease 2019” OR SARS-CoV-2) and (bamlanivimab OR etesevimab OR monoclonal antibody)

Search: **((covid 19 or covid-19) OR "coronavirus disease 2019" OR SARS-CoV-2) and (bamlanivimab OR etesevimab OR monoclonal antibody)** Sort by: **Most Recent**

("covid 19"[All Fields] OR "covid 19"[MeSH Terms] OR "covid 19 vaccines"[All Fields] OR "covid 19 vaccines"[MeSH Terms] OR "covid 19 serotherapy"[All Fields] OR "covid 19 serotherapy"[Supplementary Concept] OR "covid 19 nucleic acid testing"[All Fields] OR "covid 19 nucleic acid testing"[MeSH Terms] OR "covid 19 serological testing"[All Fields] OR "covid 19 serological testing"[MeSH Terms] OR "covid 19 testing"[All Fields] OR "covid 19 testing"[MeSH Terms] OR "sars cov 2"[All Fields] OR "sars cov 2"[MeSH Terms] OR "severe acute respiratory syndrome coronavirus 2"[All Fields] OR "ncov"[All Fields] OR "2019 ncov"[All Fields] OR (("coronavirus"[MeSH Terms] OR "coronavirus"[All Fields] OR "cov"[All Fields]) AND 2019/11/01:3000/12/31[Date - Publication]) OR ("covid 19"[All Fields] OR "covid 19"[MeSH Terms] OR "covid 19 vaccines"[All Fields] OR "covid 19 vaccines"[MeSH Terms] OR "covid 19 serotherapy"[All Fields] OR "covid 19 serotherapy"[Supplementary Concept] OR "covid 19 nucleic acid testing"[All Fields] OR "covid 19 nucleic acid testing"[MeSH Terms] OR "covid 19 serological testing"[All Fields] OR "covid 19 serological testing"[MeSH Terms] OR "covid 19 testing"[All Fields] OR "covid 19 testing"[MeSH Terms] OR "sars cov 2"[All Fields] OR "sars cov 2"[MeSH Terms] OR "severe acute respiratory syndrome coronavirus 2"[All Fields] OR "ncov"[All Fields] OR "2019 ncov"[All Fields] OR (("coronavirus"[MeSH Terms] OR "coronavirus"[All Fields] OR "cov"[All Fields]) AND 2019/11/01:3000/12/31[Date - Publication])) OR "coronavirus disease 2019"[All Fields] OR ("sars cov 2"[MeSH Terms] OR "sars cov 2"[All Fields] OR "sars cov 2"[All Fields])) AND ("bamlanivimab"[Supplementary Concept] OR "bamlanivimab"[All Fields] OR ("etesevimab"[Supplementary Concept] OR "etesevimab"[All Fields]) OR ("antibodies, monoclonal"[MeSH Terms] OR ("antibodies"[All Fields] AND "monoclonal"[All Fields]) OR "monoclonal antibodies"[All Fields] OR ("monoclonal"[All Fields] AND "antibody"[All Fields]) OR "monoclonal antibody"[All Fields]))

**Translations**

**covid 19:** ("COVID-19" OR "COVID-19"[MeSH Terms] OR "COVID-19 Vaccines" OR "COVID-19 Vaccines"[MeSH Terms] OR "COVID-19 serotherapy" OR "COVID-19 serotherapy"[Supplementary Concept] OR "COVID-19 Nucleic Acid Testing" OR "covid-19 nucleic acid testing"[MeSH Terms] OR "COVID-19 Serological Testing" OR "covid-19 serological testing"[MeSH Terms] OR "COVID-19 Testing" OR "covid-19 testing"[MeSH Terms] OR "SARS-CoV-2" OR "sars-cov-2"[MeSH Terms] OR "Severe Acute Respiratory Syndrome Coronavirus 2" OR "NCOV" OR "2019 NCOV" OR (("coronavirus"[MeSH Terms] OR "coronavirus" OR "COV") AND 2019/11/01[PDAT] : 3000/12/31[PDAT]))

**covid-19:** ("COVID-19" OR "COVID-19"[MeSH Terms] OR "COVID-19 Vaccines" OR "COVID-19 Vaccines"[MeSH Terms] OR "COVID-19 serotherapy" OR "COVID-19 serotherapy"[Supplementary Concept] OR "COVID-19 Nucleic Acid Testing" OR "covid-19 nucleic acid testing"[MeSH Terms] OR "COVID-19 Serological Testing" OR "covid-19 serological testing"[MeSH Terms] OR "COVID-19 Testing" OR "covid-19 testing"[MeSH Terms] OR "SARS-CoV-2" OR "sars-cov-2"[MeSH Terms] OR "Severe Acute Respiratory Syndrome Coronavirus 2" OR "NCOV" OR "2019 NCOV" OR (("coronavirus"[MeSH Terms] OR "coronavirus" OR "COV") AND 2019/11/01[PDAT] : 3000/12/31[PDAT]))

**SARS-CoV-2:** "sars-cov-2"[MeSH Terms] OR "sars-cov-2"[All Fields] OR "sars cov 2"[All Fields]

**bamlanivimab:** "bamlanivimab"[Supplementary Concept] OR "bamlanivimab"[All Fields]

**etesevimab:** "etesevimab"[Supplementary Concept] OR "etesevimab"[All Fields]

**monoclonal antibody:** "antibodies, monoclonal"[MeSH Terms] OR ("antibodies"[All Fields] AND "monoclonal"[All Fields]) OR "monoclonal antibodies"[All Fields] OR ("monoclonal"[All Fields] AND "antibody"[All Fields]) OR "monoclonal antibody"[All Fields]

--------------------------------------------------------------------------------

# Embase

(('covid'/exp OR covid) AND 19 OR 'covid 19'/exp OR 'covid 19' OR 'coronavirus disease 2019'/exp OR 'coronavirus disease 2019' OR 'sars cov 2'/exp OR 'sars cov 2') AND ('bamlanivimab'/exp OR bamlanivimab OR 'etesevimab'/exp OR etesevimab OR 'monoclonal antibody'/exp OR 'monoclonal antibody' OR (monoclonal AND ('antibody'/exp OR antibody)))

--------------------------------------------------------------------------------

# medRxiv

covid 19 AND (bamlanivimab)

--------------------------------------------------------------------------------
