# Supplementary figures and images for: Effects of bamlanivimab alone or in combination with etesevimab on subsequent hospitalization and mortality in outpatients with COVID-19: a systematic review and meta-analysis
Source: PeerJ. 2023 May 8;11:e15344. doi: 10.7717/peerj.15344 (PMC10174063; doi:10.7717/peerj.15344)

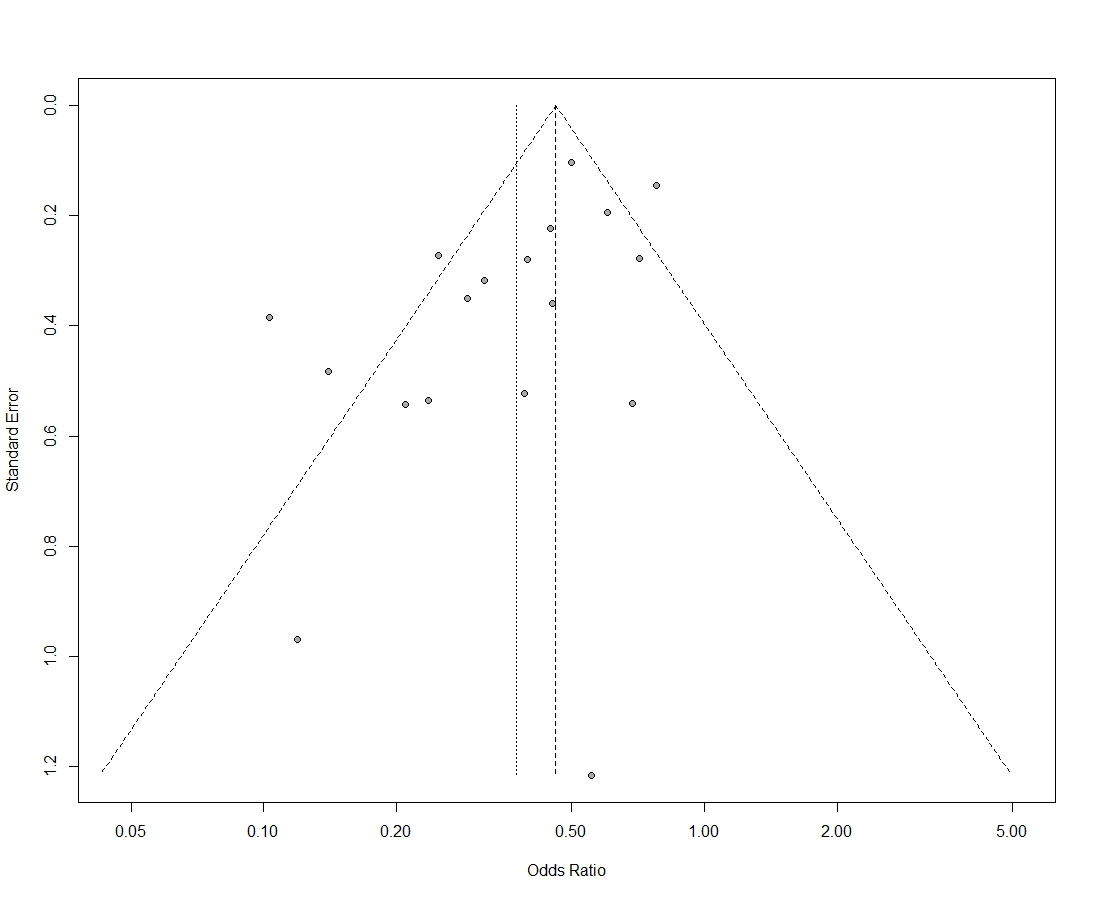

Supplement: Supplemental Information 2 [file peerj-11-15344-s002.zip › Supplementary file 2_Funnel_hospitalization_all.jpeg]

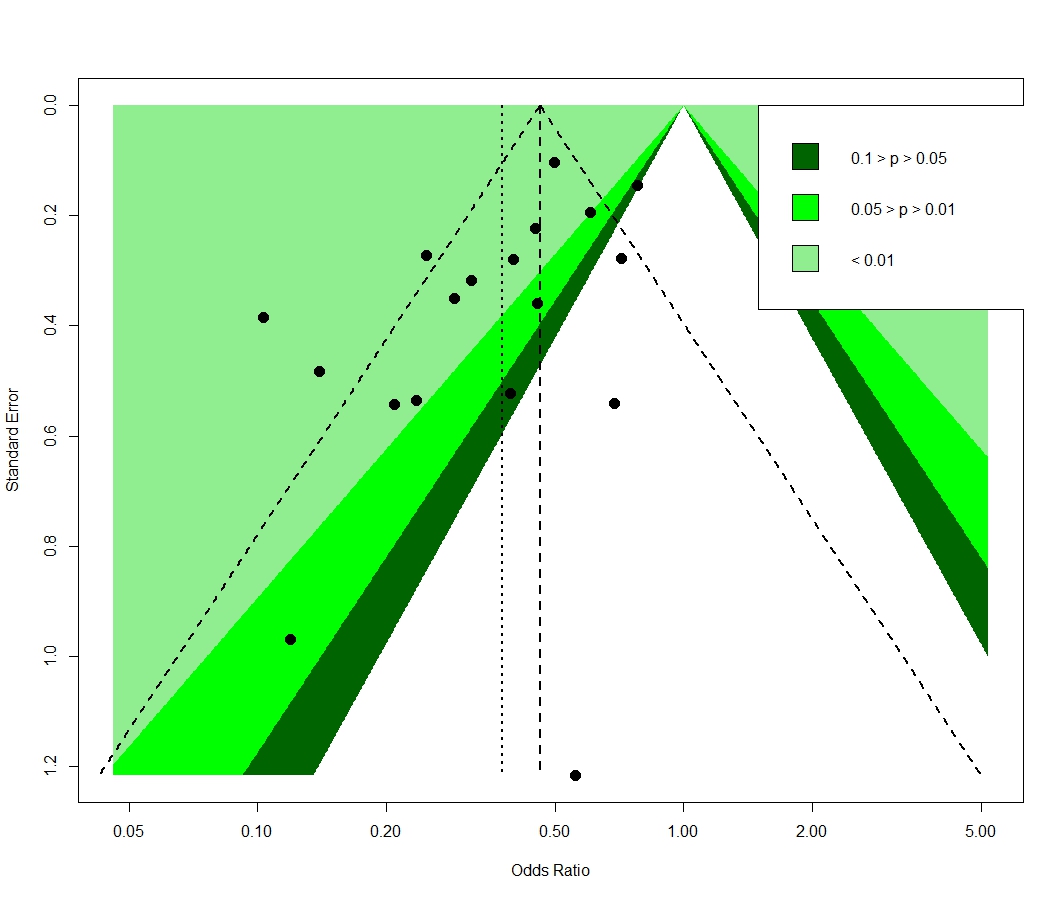

Supplement: Supplemental Information 2 [file peerj-11-15344-s002.zip › Supplementary file 3_Contour_hospitalization_all.jpeg]

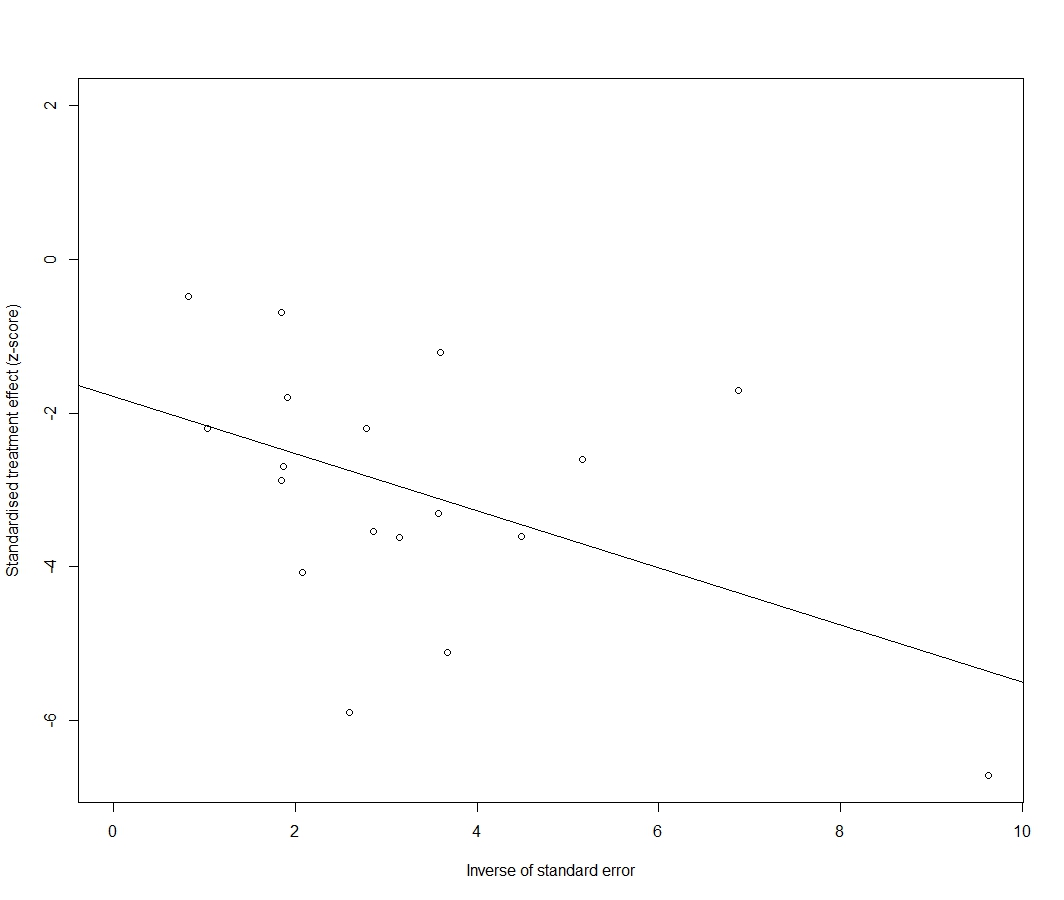

Supplement: Supplemental Information 2 [file peerj-11-15344-s002.zip › Supplementary file 4_Egger_hospitalization_all.jpeg]

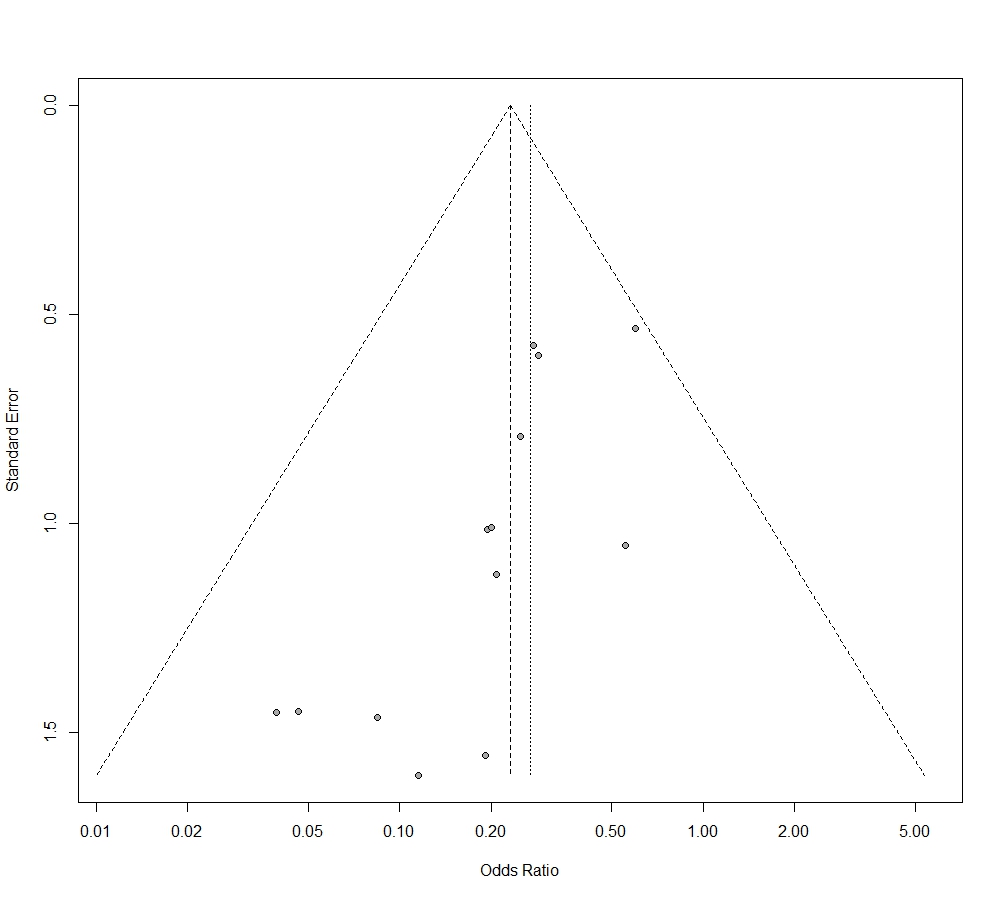

Supplement: Supplemental Information 2 [file peerj-11-15344-s002.zip › Supplementary file 5_Funnel_mortality_all.jpeg]

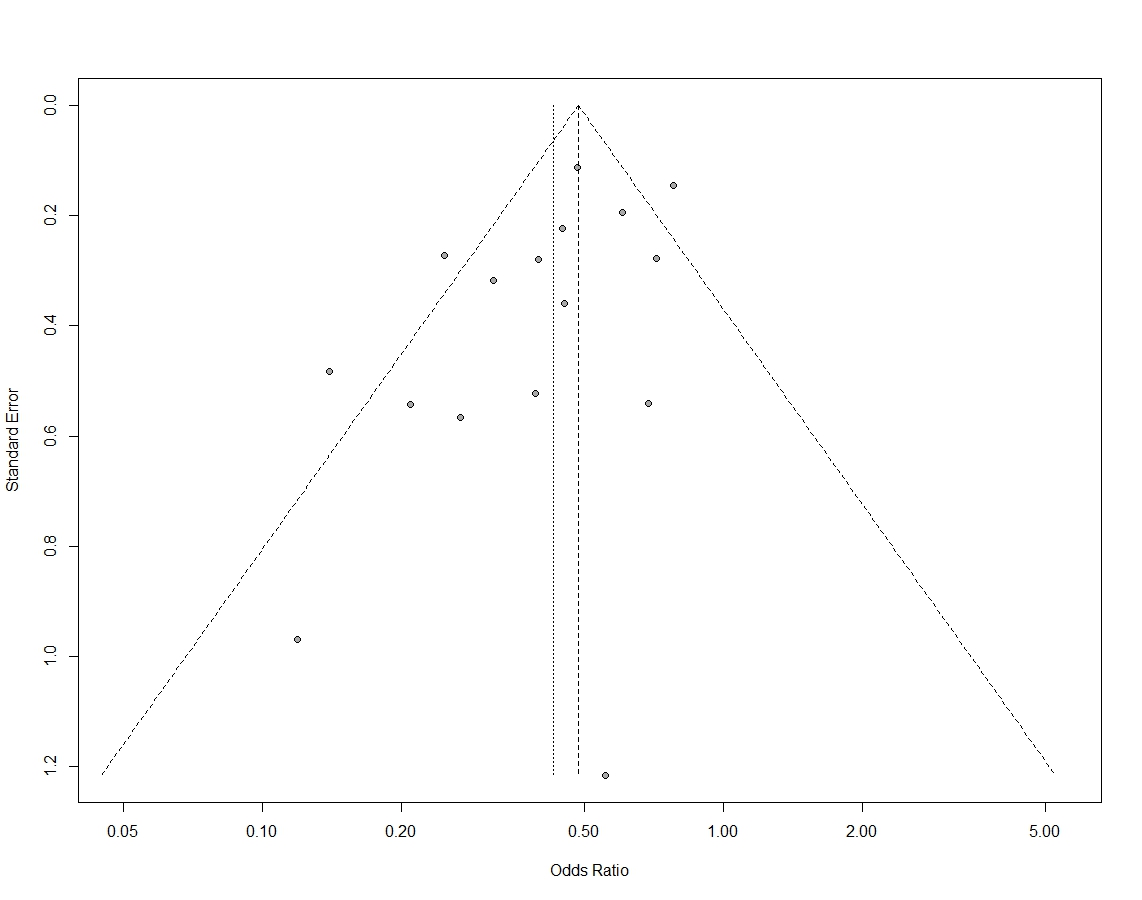

Supplement: Supplemental Information 2 [file peerj-11-15344-s002.zip › Supplementary file 6_Funnel_Hospital_B.jpeg]

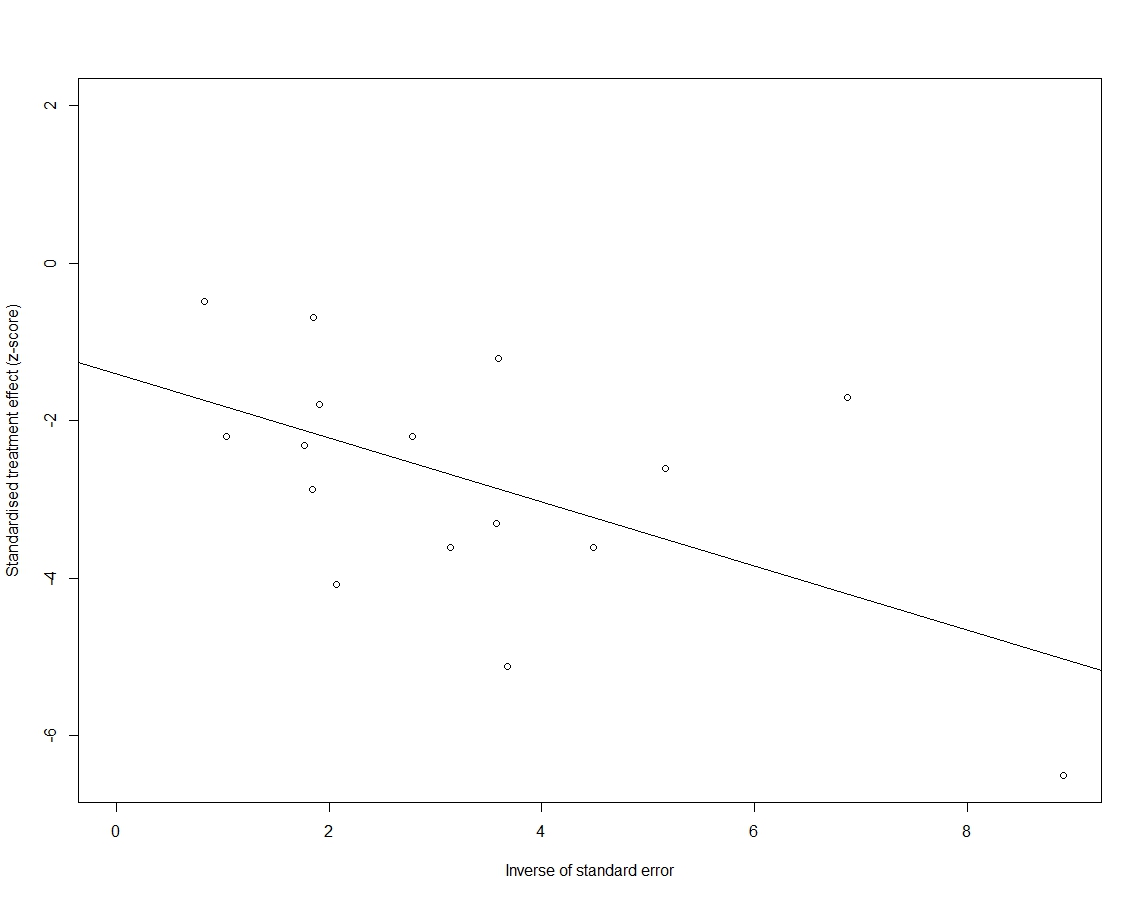

Supplement: Supplemental Information 2 [file peerj-11-15344-s002.zip › Supplementary file 7_Egger_Hospital_B.jpeg]
