# Supplementary material for: Effects of bamlanivimab alone or in combination with etesevimab on subsequent hospitalization and mortality in outpatients with COVID-19: a systematic review and meta-analysis
Source: PeerJ. 2023 May 8;11:e15344. doi: 10.7717/peerj.15344 (PMC10174063; doi:10.7717/peerj.15344)
Supplement: Supplemental Information 4 [file peerj-11-15344-s004.docx]

COVID-19 is highly contagious and early treatment contributes to lower risk of subsequent hospitalization and mortality. Medical capacity could be preserved during the pandemic. Monoclonal antibody could be administered once at emergency department or infusion center and admission is not required. Bamlanivimab is a kind of monoclonal antibody and we reviewed the efficacy and safety profiles of this drug.

There are other systematic reviews, such as Zuo et. al reviewed in J Infect 2022;84:248-288. They found a similar conclusion that bamlanivimab was effective to reduce subsequent hospitalization and mortality. Compared with their work, we enrolled studies with bamlanivimab alone or in combination with etesevimab. Therefore, more participants were enrolled and the effects of combination therapy was evaluated. Furthermore, the role of virus mutant and resistance was also discussed in our article. We believe this article is beneficial to the readers. Thank you.
